# Appropriateness of Surgical Antibiotic Prophylaxis in a Tertiary Care Teaching Hospital in Central India: A Retrospective Analysis

**DOI:** 10.7759/cureus.38844

**Published:** 2023-05-10

**Authors:** Meenalotchini P Gurunthalingam, Yogendra N Keche, Nitin R Gaikwad, Suryaprakash Dhaneria, Madhusudan P Singh

**Affiliations:** 1 Pharmacology, All India Institute of Medical Sciences, Raipur, IND; 2 Pharmacology and Therapeutics, Ruxmaniben Deepchand Gardi Medical College, Ujjain, IND

**Keywords:** guidelines, surgical site infections, surgical antibiotic prophylaxis, india, antimicrobial stewardship

## Abstract

Surgical antibiotic prophylaxis (SAP) has been a boon in the prevention of surgical site infections (SSIs). This study was conducted to assess and evaluate the selection, timing, and duration of administration of SAP and their compliance with national and international guidelines in a tertiary care teaching hospital in India.

This retrospective study included the data collected from the central records department in a tertiary care teaching hospital on major surgeries conducted between January 1, 2018, and December 31, 2018, from the departments of ENT, general surgery, orthopedic surgery, and obstetrics and gynecology. The data was analyzed for the appropriateness of their indication for SAP administration, choice, timing, and duration of antibiotics, and compliance with the American Society of Health-System Pharmacists (ASHP) and Indian Council of Medical Research (ICMR) guidelines.

Results and interpretation

Out of the total 394 case records included, only 2.53% (n = 10) of the cases were given an appropriate antibiotic. The duration of SAP was appropriate only in 6.53% (n = 24), and the timing of SAP administration was appropriate only in 50.76% (n = 204). The most commonly used antibiotic was ceftriaxone (pre-operative 58.12% (n = 229) and post-operative 43.14% (n = 170)). Major inappropriateness was observed in the selection of antibiotics which may be attributed to the non-availability of cefazolin in the institute. The inappropriateness of the duration of the SAP may be attributed to the extra precautions taken by the treating physicians to prevent SSIs. The overall compliance of the surgical cases with respect to the ASHP and ICMR guidelines was less than 1%.

Conclusion

This study identified the lacuna between the guidelines for SAP and the clinical application of the same. It also identified the areas where quality improvement was needed which can be improved by implementing antimicrobial stewardship, especially the choice and the duration of SAP administration.

## Introduction

Any patient may develop a surgical site infection (SSI) after surgery, regardless of whether it is a major or minor surgery [1]. These infections may range from a superficial incision site abscess to a complex infection involving multiple organs leading to sepsis and even mortality of the patient. SSIs account for about 31% of hospital-acquired infections. SSIs result in pain, discomfort, increased hospital stay, increased cost of treatment, loss of income, more interventions on the patients, increased readmission rates, increased healthcare costs, decreased quality of life for the patient, and even mortality [2,3]. Hence, the prevention of SSI is of utmost importance, and it has become a universal measure of quality in hospital-based surgical practice.

Appropriate use of surgical antibiotic prophylaxis (SAP) has proven to control SSIs and reduce morbidity and mortality [4]. However, inappropriate use of SAP is associated with the development of antibiotic resistance, resistant pathogens causing superinfections, increased toxicity, and an unnecessary financial burden. To overcome these shortcomings, the American Society of Health-System Pharmacists (ASHP) [5,6], WHO [7], and the Indian Council for Medical Research (ICMR) [4] have laid out guidelines to be followed while prescribing perioperative antibiotic prophylaxis. Despite the availability of the above guidelines, compliance with these guidelines is still low in many countries. About 30-50% of antibiotics used in hospitals are prescribed for surgical prophylaxis, and 30-90% of this SAP is inappropriate [8]. Therefore, this study was conducted to assess the perioperative use of antibiotics in patients undergoing surgical procedures and their compliance with the guidelines in a tertiary care teaching hospital.

## Materials and methods

The aim of this study was to analyze antimicrobial usage in surgical prophylaxis in a tertiary care hospital. The primary objective was to evaluate the selection, timing, and duration of administration of prophylactic antibiotics among surgical patients, and the secondary objective was to evaluate the compliance of SAP using ASHP and ICMR guidelines for SAP.

This study was a retrospective longitudinal cohort study conducted in a tertiary care teaching hospital in Raipur, Chhattisgarh, India. After obtaining ethical clearance from the Institute Ethics Committee, the surgical data for the study was collected from the central records department of the teaching hospital. The inclusion and exclusion criteria for the study are shown in Table 1.

**Table 1:** Inclusion and exclusion criteria

Inclusion criteria	Exclusion criteria
All patients >18 years of age who underwent major elective surgeries between January 1, 2018, and December 31, 2018, in the departments of otorhinolaryngology, general surgery, orthopedic surgery, and obstetrics and gynecology surgeries with clean, clean-contaminated, and contaminated wounds	Patients <18 years of age, surgeries with dirty wounds, emergency surgeries, therapeutic and other non-surgical prophylactic uses

**Figure 1:**
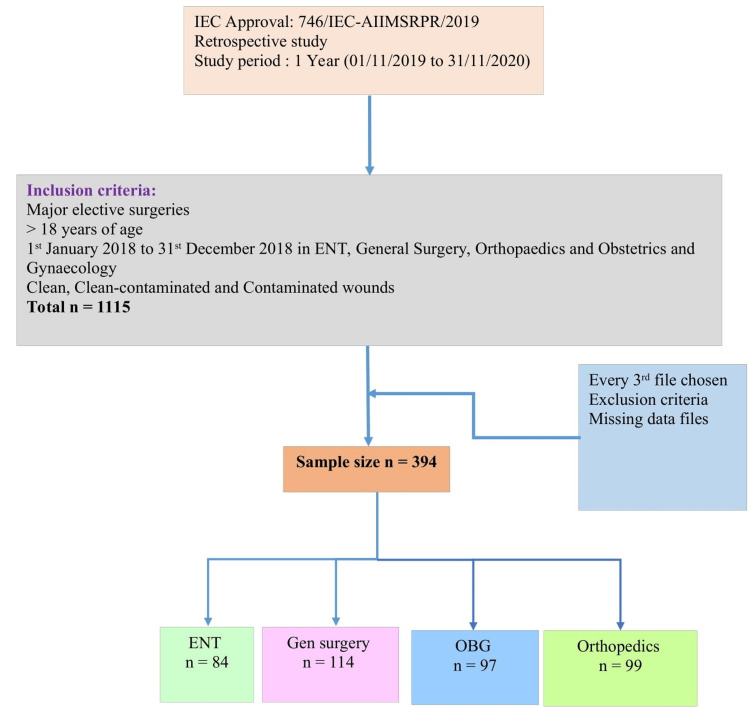
Flowchart of the selection of files ENT: otorhinolaryngology; gen surgery: general surgery; OBG: obstetrics and Gynecology; ortho: orthopedic surgery

A total of 1115 major elective surgeries have taken place in the departments of ENT (n = 240), general surgery (n = 335), obstetrics and gynecology (n = 281), and orthopedic surgery (n = 255) during the period of January 1, 2018, to December 31, 2018. The sample size was calculated to be 394 (including 20% files with missing data) based on a study on adherence to SAP in a tertiary care hospital in India [9], where the prevalence of inappropriateness to SAP was found to be 30.8%. Every third file was chosen for analysis after excluding those with missing data and those that fulfilled the exclusion criteria. The flowchart of the selection of files is shown in Figure 1. The relevant data from the patient's medical records were collected on the basis of the four sections of the data abstraction format which include patient demographic data (patient initials, age, and sex), surgical data (class and type of surgery, time of incision, duration of surgery, wound class, and duration of hospital stay), SAP usage data (antibiotic name, dose, frequency, pre-operative administration time relative to incision, and duration of prophylactic administration), and appropriate SAP usage assessment (indication, selection, duration, and timing) [10].

## Results

The median age of the patients was 39 years and 57.1% were female patients and 42.9 were male patients. About 56.14% of the general surgery cases had clean wounds, 100% of the obstetrics and gynecology cases had clean-contaminated wounds, and 6.06% of the orthopedic cases had contaminated wounds. About 21.92% and 58.77% of the general surgery cases took place for less than one hour and between one and two hours, respectively. About 33.33% of the orthopedic surgeries had taken place between two and four hours, and 10.71% of the ENT surgeries had taken place for more than four hours. The maximum length of hospital stay was observed in the department of orthopedic surgery with an average of 12 days, and the least length of hospital stay was observed in the department of general surgery with a mean value of 6.7 days.

The most common antimicrobial used in the department of ENT was intravenous amoxicillin + clavulanic acid, both pre-operatively (79.76% (n = 67)) and post-operatively (78.57% (n = 66)). Post-operatively, intravenous metronidazole has also been administered in 39.28% (n = 33) of patients. Intravenous vancomycin has been used in 1.19% (n = 1) of patients post-operatively.

In the department of general surgery, intravenous ceftriaxone has been used in 94.73% (n = 108) of patients pre-operatively, whereas post-operatively, it has been used in 61.40% (n = 70) of patients. Intravenous amoxicillin + clavulanic acid has been used in 0.87% (n = 1) of patients pre-operatively and in 19.29% (n = 22) of patients post-operatively.

In the department of obstetrics and gynecology, intravenous ceftriaxone has been used in 98.96% (n = 96) and 88.85% (n = 86) of patients pre-operative and post-operative, respectively. Intravenous metronidazole has been used in 83.50% (n = 81) of patients post-operatively. Other than these, intravenous gentamicin and intravenous amikacin have been used in 8.24% (n = 8) of patients post-operatively. Intravenous piperacillin-tazobactam has been used in 3.09% (n = 3) of patients post-operatively.

In the department of orthopedic surgery, Inj. amikacin has been used in 93.93% (n = 93) and 98.98% (n = 98) of patients pre-operatively and post-operatively, respectively. Inj. cefuroxime has been used in 75.75% (n = 75) and 83.83% (n = 83) of patients pre-operatively and post-operatively, respectively. Inj. ceftriaxone has been used in 17.17% (n = 17) of patients pre-operatively and 7.07% (n = 7) of patients post-operatively. Similarly, Inj. piperacillin-tazobactam has been used in 17.17% (n = 17) of patients pre-operatively.

About 20.17% and 74.56% of general surgery cases had prophylaxis administration for one day and between two and seven days, respectively. About 27.27% and 8.08% of the orthopedic surgeries had been administered prophylaxis between 8 and 14 days and for more than 15 days, respectively.

**Figure 2:**
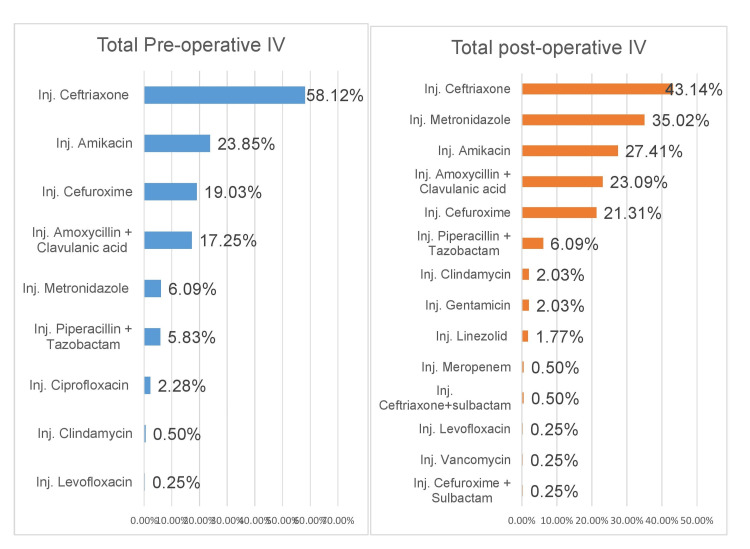
Total pre-operative and post-operative antibiotic usage

The total antibiotic usage in the pre-operative and post-operative periods in all four departments is shown in Figure 2.

The ASHP 2013 and ICMR 2017 guidelines were similar with respect to all the indicators except for the selection of SAP. According to the ASHP 2013 guidelines, 21.82% of cases were administered broader SAP, whereas it was 22.33% according to the ICMR 2017 guidelines. Similarly, 78.38% of the overall cases were administered inappropriate SAP according to ASHP 2013 guidelines, whereas it was 74.86% according to the ICMR 2017 guidelines (Table 2).

**Table 2:** Comparison of ASHP 2013 and ICMR 2017 guidelines Adequate/compliant: if the drug(s) used are recommended in the guidelines Narrow: when only one of the drugs recommended by the guideline is used Broader: when extra drug along with those recommended is added to the recommended guideline Unrelated: if the drug is not recommended in the guidelines or if it is regardless of its spectrum of coverage

Compliance indicators^[10]^		Guidelines	
		ASHP 2013	ICMR 2017
Indication		350 (88.83%)	350 (88.83%)
Selection of SAP	Adequate	10 (2.53%)	10 (2.53%)
	Narrow	0 (0)	0 (0%)
	Broader	86 (21.82%)	88 (22.33%)
	Inappropriate	297 (75.38%)	295 (74.87%)
Duration of SAP		24 (6.09%)	24 (6.09%)
Timing of SAP		204 (51.77%)	204 (51.77%)

Two surgeries were indicated for intra-operative re-dosing as per the guidelines. However, the SAP re-dosing was done in only one of the cases (Table 3).

**Table 3:** List of surgeries indicated for intra-operative redosing of SAP

S.no	Department	Surgery	Antibiotics used pre-operatively	Half-life	Time of pre-operative SAP	Start of surgery	Duration of surgery	Re-dosing
1.	ENT	Wide local excision + marginal mandibulectomy + split skin graft + supra omohyoid neck dissection (right)	Intravenous amoxicillin + clavulanate intravenous metronidazole	1-2 hours 6-8 hours	6.00 am	10.00 am	5 hours	Given
2.	Orthopedic surgery	Spinal surgery	Intravenous piperacillin-tazobactam 4.5 gm	0.7-1.2 hours	9.00 am	11.00 am	6 hours	Not given

Out of 394 cases, only three cases had 100% compliance with respect to all indicators. All three cases underwent cholecystectomy, where intravenous. ceftriaxone was administered 60 minutes before the incision for less than 24 hours.

## Discussion

In our study, we used the "ASHP Clinical Practice Guidelines for Antimicrobial Prophylaxis" in Surgery published in 2013 and the "Treatment Guidelines for Antimicrobial Use in Common Syndromes" published by the ICMR in 2017 to assess the compliance of SAP usage. The ASHP clinical practice guidelines for antimicrobial prophylaxis in surgery are the most commonly used guidelines to evaluate SAP adherence. In addition to the SAP usage adherence to the ASHP 2013 guidelines, we have evaluated the adherence of SAP usage with regards to the ICMR 2017 guidelines, as we wanted to assess the adherence of SAP usage in an Indian setup using Indian guidelines.

In our study, we observed that compliance with the guidelines for indication of the SAP was 88.83%. SAP is usually not indicated in clean surgeries, except for those with prostheses [6]. However, SAP was administered even in clean surgeries due to apprehension of SSI likely to occur due to probable breach of asepsis in the operative area and post-operative wards. It may also be due to inadequate awareness regarding the recent updates of guidelines [11].

In our study, the timing of SAP was appropriate in 51.77% of the total cases. This is almost similar to the observation made by Chandrasekaran et al. [12]. In our study, the compliance rate with regard to the timing of SAP was observed to be highest in the department of obstetrics and gynecology (68.04%) followed by general surgery (61.0%). Of the total 84 ENT cases, only 10 received the SAP within the stipulated 60 minutes prior to the incision. It was observed that the SAP was administered in the morning by the nursing staff regardless of the time of surgery. This was similar to a study conducted by Shankar et al. [13]. This may be due to a lack of communication between the prescribing surgeon or physician and the nursing staff or a lack of knowledge regarding the time of administration of SAP.

In our present study, the choice of SAP was inappropriate in 75.38% of the cases. The drug of choice for SAP given in the ASHP and ICMR 2017 guidelines is cefazolin. However, cefazolin was not administered in a single case. This may be attributed to the non-availability of cefazolin at the institute during the time when the surgeries took place (information received from the central pharmacy). As mentioned by Kakkar et al., the non-availability of cefazolin in the institute may be attributed to the poor availability of essential drugs like cefazolin in lower- and middle-income countries [14]. Instead of cefazolin, the third-generation cephalosporin, ceftriaxone was administered in the majority of cases. This was similar to the studies that were conducted in Ethiopia, Malaysia, Riyadh, Indonesia, and Pakistan [10,15-18]. The use of broad-spectrum antibiotics is not preferred and is not advised in the national and international guidelines due to the fact that it may lead to the emergence of drug-resistant microorganisms [4,6]. In addition to ceftriaxone, amikacin, cefuroxime, amoxicillin-clavulanate, and metronidazole were also used. In all cholecystectomy cases, intravenous ceftriaxone was administered as per the guidelines. The choice of SAP was appropriate, i.e., completely compliant with the guidelines, in 2.79% of the total cases. Eight cases from general surgery and three cases from ENT were administered adequate choice of SAP. This is far below the appropriateness seen in studies conducted in Greece (70%), Australia (92.5%), and Pakistan (57.33%) [19-21].

The reason why ceftriaxone was widely used may be due to its easy availability compared to cefazolin. It has a long half-life (eight hours), and it has been observed that the plasma level of ceftriaxone was above the minimum inhibitory concentration (8.5-55.2 mg/dl) of most of the microorganisms responsible for post-operative infections. Hence, there is no need for another dose intra-operatively. One of the studies proposed the assumption that due to the high protein binding property of ceftriaxone, the serum level of the drug is maintained even after excessive blood loss [22].

On the other hand, the use of ceftriaxone is not recommended because it is a "Watch" group antibiotic as per WHO’s AWaRe classification, which means that ceftriaxone has a higher potential for resistance development and it is also listed in the WHO's highest priority, critically important antimicrobials list [23,24]. It is highly concentrated in the biliary tract causing collateral damage to the gut flora and making the patient susceptible to *Clostridium difficile* infection. Ceftriaxone is less effective against methicillin-sensitive *S. aureus* compared to cefazolin [25].

According to the guidelines, an ideal prophylactic antibiotic should be active against the microorganisms that are most likely to contaminate the site of surgery without any adverse effects and should be administered at the right time to maintain an adequate concentration of the drug at the site of surgery and should be used for the shortest period of time [6]. However, in our study, we observed that linezolid, which is a "Reserve" group antibiotic, was being used for an average of five days. Similarly, the "Watch" group antibiotics other than the cephalosporins were being used for a longer duration than the prescribed 24 hours postoperatively. Among the "Watch" group antibiotics, meropenem was used for 7.5 days, piperacillin-tazobactam was used for 6.5 days, vancomycin was used for 7 days, and levofloxacin was used for an average of 9 days. This unnecessary exposure of the patients to the "Watch" and "Reserve" group antibiotics increases the chances of the development of antimicrobial resistance, increases adverse effects, increases the length of hospital stay, and increases the cost of treatment.

As we observed that there was increased use of "Watch" and "Reserve" group antibiotics, we analyzed the diagnosis, surgical procedure, and class of wounds where such antibiotics were used. We observed that the "Reserve" group antibiotic, linezolid, was used in 1.7% (7) of the total patients which included six clean-contaminated and one contaminated wound. Similarly, among the "Watch" group antibiotics, meropenem was used in 0.50% (2) of the cases, both belonging to clean-contaminated wounds. Vancomycin in 0.25% (one) of the cases, belonged to the clean wound. Piperacillin-tazobactam was used in 6.09% (24) of patients, among which six cases were clean wounds, 13 were clean-contaminated wounds, and five were contaminated wounds. Levofloxacin was used in 0.50% (two), one case each in clean-contaminated and contaminated wounds. Ciprofloxacin was used in 2.53% (10) of the total patients, among which seven were clean-contaminated and three were contaminated wounds.

The complete compliance rate with the ASHP guidelines was observed to be 0.76% in our present study. The inappropriateness was observed to be greatest with the choice of antibiotic and duration of SAP administration. This was consistent with the study conducted in Ethiopia, Riyadh, Indonesia, and Pakistan [10,16-18]. In our study, we checked for compliance with the ICMR 2017 "Treatment guidelines for antimicrobial use in common syndromes." According to the ICMR 2017 guidelines, the drug of choice for clean wounds is either cefuroxime or cephalexin, whereas according to the ASHP guidelines, cefazolin is the drug of choice for the majority of surgeries, including clean and clean-contaminated surgeries [4,6]. This difference was observed in only two cases. It was observed that according to ASHP guidelines, the inappropriateness was 75.38% (297 cases), whereas according to the ICMR 2017, it was observed to be 74. 87% (295 cases). In our study, the compliance of all the other indicators for SAP appropriateness was observed to be similar according to both guidelines.

Intra-operative administration of SAP has been advised for surgeries that take place for more than two half-lives of the antibiotic administered pre-operatively and surgeries where the blood loss during surgery is more than 1500 ml [4,6]. In our present study, two surgeries, one in ENT and one in orthopedics, were indicated for intra-operative SAP administration. In one case of the ENT, intravenous amoxicillin clavulanate was used pre-operatively for SAP, and it was re-dosed because the duration of surgery was longer than two half-lives of this drug. In an orthopedic surgery that took place for six hours, intravenous piperacillin-tazobactam was administered pre-operatively; even though the total duration of the surgery along with the pre-operative SAP administration time was longer than two half-lives of piperacillin-tazobactam, intra-operative re-dosing was not done. No other studies have given a clear account of intra-operative re-dosing in their observations. The reason for which intraoperative re-dosing was not done may be due to incomplete maintenance of the intra-operative anesthetic and nursing notes, unawareness regarding redosing, or the extensive pre-operative and post-operative antibiotic coverage.

The non-compliance with the duration of SAP administration may be due to unawareness regarding the recommended duration of SAP, or the belief that extra precautions were taken by the treating physicians to avoid SSIs by administering SAP for a longer duration [18,26,27], and/or due to insecurity regarding the legal pursuits in case if any complications arise due to the administration of shorter duration of SAP [28].

This can be overcome by providing “SAP kits,” which will contain the right choice of antibiotic at the right dose for the right duration. For example, for a patient undergoing modified radical mastectomy with axillary lymph node sampling, where SAP is indicated, three doses of injection, Cefazolin 2 gm, can be made as a kit with proper instructions. The instructions should include that one dose of cefazolin should be administered pre-operatively within 60 minutes prior to the incision, and another dose of the drug should be administered post-operatively if needed. The extra dose should be reserved for intra-operative redosing if the surgery extends for more than four hours. No extra dose of the antimicrobial should be administered once the kit is empty. The health care worker should be made responsible to record the time of administration of each dose appropriately. The instructions could be displayed in the nursing stations so that the nursing staff who administer the SAP are made acquainted or conscious about the dose, time of administration of SAP, and also the duration of SAP. This approach has been successful in studies in Spain and France [29,30].

In our study, emergency surgeries and dirty wound surgeries were not taken into account for comparing adherence with guidelines because emergency surgeries require multiple levels of care [13]. This may lead to the choice of multiple antibiotics with a broad-spectrum, inappropriate timing of SAP administration, and probable longer duration of SAP administration. The dirty wound surgeries are those that are already infected. Hence, there will be a need for adequate treatment of the wounds prior to the surgery rather than a prophylactic approach for the prevention of SSIs in such cases [6].

This study provides evidence that there is a wide gap between the guidelines and the practice of SAP. Hence, there is a need to improve the practice of SAP in accordance with the available national and international guidelines for SAP to prevent the emergence of antibiotic resistance without compromising the safety of patients.

Strengths

Our study is the first of its kind to check for the compliance of SAP with the ICMR guidelines. The results of our study suggest that the guidelines of ASHP and ICMR are comparable and equally good for SAP prescription. Our study recorded the cases where the re-dosing was done and not done. The data in our study were analyzed against standard guidelines. This study helped to identify the areas for antimicrobial stewardship interventions and will also help in the development of antibiotic policy at the institute.

Limitations

Since it is a retrospective study, information regarding the missing data could not be obtained. In this study, the factors influencing the incidence of SSIs like body mass index, smoking status, alcohol intake, baseline nutritional status of the patients, medical co-morbid conditions like severe anemia and diabetes mellitus, and the use of medications like immunosuppressants and steroids could not be taken into account due to inconsistent information. These factors may be the influencing factors for prolonged SAP administration. Prolonged SAP administration may be the cause of SSIs; however, we were unable to assess SSIs as the incidence of SSIs can be better determined in a prospective study.

## Conclusions

In the current study, the most common antimicrobial used was Inj. ceftriaxone. The results of this study suggest that the guidelines of ASHP and ICMR are comparable and are equally good for SAP prescription. The major inappropriateness was recorded with respect to the duration of SAP administration and the choice of antibiotic administered. Improvement in these parameters through the implementation of antimicrobial stewardship in the institute, and quality improvement strategies like regular periodic training of healthcare workers, regular audits of SAP prescriptions, and the display of pamphlets and posters will lead to an overall improvement in SAP compliance. This will further help in the prevention of the development of antimicrobial resistance, thereby ensuring better patient care. These measures will also help improve the knowledge and practice of the surgical residents in prescribing the SAP.

## References

[1] Garner BH, Anderson DJ (2016). Surgical site infections: an update. Infect Dis Clin North Am.

[2] Pedroso-Fernandez Y, Aguirre-Jaime A, Ramos MJ, Hernández M, Cuervo M, Bravo A, Carrillo A (2016). Prediction of surgical site infection after colorectal surgery. Am J Infect Control.

[3] Graves EJ, Kozak LJ (1996). National hospital discharge survey: annual summary, 1996. Vital Health Stat.

[4] (2023). ICMR treatment guidelines for antimicrobial use in common syndromes. Accessed on February 28. https://www.icmr.nic.in/sites/.../guidelines/treatment_guidelines_for_antimicrobial.pdf..

[5] (2023). therapeutic-guidelines-antimicrobial-prophylaxis-surgery.pdf.. https://www.ashp.org/-/media/assets/policy-guidelines/docs/therapeuticguidelines/therapeutic-guidelines-antimicrobial-prophylaxis-surgery.ashx.

[6] Bratzler DW, Dellinger EP, Olsen KM (2013). Clinical practice guidelines for antimicrobial prophylaxis in surgery. Am J Health Syst Pharm.

[7] (2023). Global guidelines for the prevention of surgical site infection- WHO 2018. https://www.who.int/infection-prevention/publications/ssi-guidelines/en/.

[8] Dettenkofer M, Forster DH, Ebner W, Gastmeier P, Rüden H, Daschner FD (2002). The practice of perioperative antibiotic prophylaxis in eight German hospitals. Infection.

[9] Jaggi N, Nirwan P, Chakraborty M (2018). Adherence to surgical antibiotic prophylaxis guidelines in an Indian tertiary care hospital. J Patient Saf Infect Control.

[10] Alemkere G (2018). Antibiotic usage in surgical prophylaxis: a prospective observational study in the surgical ward of Nekemte referral hospital. PLoS One.

[11] Hassan S, Chan V, Stevens J, Stupans I (2021). Factors that influence adherence to surgical antimicrobial prophylaxis (SAP) guidelines: a systematic review. Syst Rev.

[12] Chandrasekaran K, Saeed K, Gandhiraj D, Mohanta GP, Rajasekaran A (2016). Study on adherence to prophylactic antimicrobials use guidelines in surgical wards of an Indian private corporate hospital. Int J Pharma Res Allied Sci.

[13] Shankar R (2018). Implementation of the WHO Surgical Safety Checklist at a teaching hospital in India and evaluation of the effects on perioperative complications. Int J Health Plann Manage.

[14] Kakkar AK, Shafiq N, Malhotra S (2021). Cefazolin shortages in the developing world: the same, but different too. Clin Infect Dis.

[15] Oh AL, Goh LM, Nik Azim NA, Tee CS, Shehab Phung CW (2014). Antibiotic usage in surgical prophylaxis: a prospective surveillance of surgical wards at a tertiary hospital in Malaysia. J Infect Dev Ctries.

[16] Ahmed N, Balaha M, Haseeb A, Khan A (2022). Antibiotic usage in surgical prophylaxis: a retrospective study in the surgical ward of a governmental hospital in Riyadh region. Healthcare (Basel).

[17] Herawati F, Yulia R, Hak E, Hartono AH, Michiels T, Woerdenbag HJ, Avanti C (2019). A retrospective surveillance of the antibiotics prophylactic use of surgical procedures in private hospitals in Indonesia. Hosp Pharm.

[18] Khan Z, Ahmed N, Rehman AU, Khan FU, Saqlain M, Martins MA, Rahman H (2020). Audit of pre-operative antibiotic prophylaxis usage in elective surgical procedures in two teaching hospitals, Islamabad, Pakistan: an observational cross-sectional study. PLoS One.

[19] Rafati M, Shiva A, Ahmadi A, Habibi O (2014). Adherence to American society of health-system pharmacists surgical antibiotic prophylaxis guidelines in a teaching hospital. J Res Pharm Pract.

[20] Ierano C, Thursky K, Marshall C (2019). Appropriateness of surgical antimicrobial prophylaxis practices in Australia. JAMA Netw Open.

[21] Satti MZ, Hamza M, Sajid Z, Asif O, Ahmed H, Zaidi SM, Irshad U (2019). Compliance rate of surgical antimicrobial prophylaxis and its association with knowledge of guidelines among surgical residents in a tertiary care public hospital of a developing country. Cureus.

[22] Geroulanos S, Marathias K, Kriaras J, Kadas B (2001). Cephalosporins in surgical prophylaxis. J Chemother.

[23] (2023). WHO model list of essential medicines. https://www.who.int/medicines/publications/essentialmedicines/en/.

[24] (2023). Critically important antimicrobials for human medicine. http://apps.who.int/iris/bitstream/handle/10665/255027/9789241512220-eng.pdf;jsessionid=003679F19673AC9743C39CA8E6085A18?sequence=1.

[25] Sullivan SA, Smith T, Chang E, Hulsey T, Vandorsten JP, Soper D (2007). Administration of cefazolin prior to skin incision is superior to cefazolin at cord clamping in preventing postcesarean infectious morbidity: a randomized, controlled trial. Am J Obstet Gynecol.

[26] Knox MC, Edye M (2016). Adherence to surgical antibiotic prophylaxis guidelines in New South Wales, Australia: identifying deficiencies and regression analysis of contributing factors. Surg Infect (Larchmt).

[27] Karaali C, Emiroglu M, Esin H (2020). Assessment of prophylactic antibiotic usage habits of the general surgeons in Turkey. J Infect Dev Ctries.

[28] Rohrer F, Maurer A, Noetzli H, Gahl B, Limacher A, Hermann T, Bruegger J (2021). Prolonged antibiotic prophylaxis use in elective orthopaedic surgery - a cross-sectional analysis. BMC Musculoskelet Disord.

[29] Mondelo García C, Gutiérrez Urbón JM, Pérez Sanz C, Martín Herranz MI (2018). Auditing and improving surgical antibiotic prophylaxis. Surg Infect (Larchmt).

[30] Carlès M, Gindre S, Aknouch N, Goubaux B, Mousnier A, Raucoules-Aimé M (2006). Improvement of surgical antibiotic prophylaxis: a prospective evaluation of personalized antibiotic kits. J Hosp Infect.

